# Functionality-Driven Optimization of Green Ultrasound-Assisted Extraction of Antioxidant Compounds from Edible Brown Algae

**DOI:** 10.3390/md23120469

**Published:** 2025-12-07

**Authors:** Carolina Padrón-Sanz, Samanta García-Oms, Javier Pacheco-Juárez, Lorena Pasquali, Dolores Cejalvo-Lapeña

**Affiliations:** 1 Centro de Investigación Traslacional San Alberto Magno (CITSAM), Universidad Católica de Valencia, 46001 Valencia, Spain; dolores.cejalvo@ucv.es; 2 Escuela de Doctorado, Universidad Católica de Valencia, 46001 Valencia, Spain; samanta.garcia@avantorsciences.com; 3 Instituto Universitario de Estudios Ambientales y Recursos Naturales (i-UNAT), Universidad de Las Palmas de Gran Canaria, 35017 Las Palmas, Spain; javier.pacheco@ulpgc.es; 4 Primolab S.r.l., 31027 Spresiano, Italy; pasqualilorena2@gmail.com

**Keywords:** antioxidants, ultrasound-assisted extraction, brown algae, polyphenols, pigments, UHPLC-MS/MS, HPLC-Fl

## Abstract

The extraction of antioxidant compounds from brown macroalgae is of growing industrial interest; however, the weak correlation often observed between polyphenol content and antioxidant activity challenges the conventional strategy of optimizing only extraction yield. This study introduces, for the first time in brown macroalgae, a functionality-driven optimization approach in which ultrasound-assisted extraction (UAE) conditions are optimized based on antioxidant activity as the primary response variable, rather than compound concentration. A green UAE process was developed and optimized for four edible brown algae (*Himanthalia elongata*, *Eisenia bicyclis*, *Sargassum fusiforme*, and *Laminaria ochroleuca*), considering algae amount, solvent type and concentration, extraction time, ultrasound power, and temperature. The optimized extracts achieved 69.17–94.68% DPPH inhibition, together with high antioxidant capacity supported by ORAC (18.63–491.30 μmol TE g^−1^ DW) and FRAP (1.24–87.65 µmol Fe^+2^ g^−^^1^ DW) values, identifying *E. bicyclis* and *H. elongata* as the most promising species. Chromatographic analyses confirmed the presence of phlorotannins and carotenoid pigments such as fucoxanthin as the main contributors to antioxidant activity. Overall, this work validates a functionality-driven UAE optimization strategy for efficiently maximizing antioxidant activity in brown algal extracts.

## 1. Introduction

Research on compounds with antioxidant activity has experienced exponential growth in recent decades due to their beneficial properties and multiple applications in promising sectors such as the pharmaceutical, food, and cosmetic industries [1,2,3]. Moreover, the search for these compounds from natural sources has become particularly relevant, as natural extracts are generally more accepted by consumers and avoid the undesirable side effects associated with some synthetic antioxidants used in food products [4].

In this context, the development and optimization of extraction methods for antioxidant compounds from marine algae have attracted increasing attention, since these organisms have undergone adaptive processes that enable them to produce secondary metabolites with strong antioxidant potential. In particular, brown and red algae are among the richest sources of bioactive antioxidant compounds such as polyphenols, pigments, and carbohydrates [5,6,7].

Accordingly, several extraction methodologies have been proposed to maximize the recovery of these compounds, contributing not only to the isolation of high-value biomolecules but also to the sustainable valorization of algal biomass [8]. Traditional techniques, such as solid–liquid extraction, maceration, or cold percolation, have proven to be effective and easily scalable to the industrial level. However, they often require large volumes of toxic organic solvents, prolonged heating, or complex sequential procedures to recover different classes of bioactive compounds [9,10]. Consequently, the main current challenge is to develop extraction methods that minimize solvent consumption, reduce extraction time and energy demand, and streamline the extraction workflow, ultimately achieving environmentally sustainable approaches without compromising efficiency.

One methodology that fulfills these criteria, and is therefore aligned with the principles of green chemistry, is ultrasound-assisted extraction (UAE), which has been successfully applied for the recovery of bioactive compounds from various natural matrices [11]. UAE has gained wide acceptance for isolating algal bioactives because it is faster, more environmentally sustainable, scalable to pilot levels, and highly efficient in recovering compounds of interest such as polyphenols, carbohydrates, and pigments [12,13,14,15]. These properties make UAE a suitable green alternative to conventional extraction methods.

However, as in other extraction approaches, optimization strategies are frequently oriented toward achieving the highest possible concentration of the target compounds. This assumes a direct correlation between compound concentration and biological activity. In the case of antioxidant activity, it is often presumed that higher concentrations of antioxidant molecules (e.g., polyphenols) lead to higher overall activity. Surprisingly, for brown algae this correlation is not always observed and may arise from multiple factors: As reported in previous studies, the antioxidant performance of algal polyphenols is strongly structure-dependent: the number and relative position of hydroxyl groups influence both radical-scavenging and metal-chelating ability [16,17,18]. Moreover, co-extracted bioactive molecules such as carotenoid pigments can contribute significantly to the overall activity [19]. In addition, antioxidant capacity may be driven by a few highly active but less abundant compounds rather than by total polyphenol concentration [20].

For all these reasons, unlike previous extraction optimization studies that primarily focused on maximizing yield or TPC, the present work systematically uses antioxidant activity as the response variable to guide the optimization of UAE conditions. The optimized extracts are subsequently subjected to detailed UHPLC-MS/MS and HPLC-FL analyses, enabling the identification of compounds contributing to the observed antioxidant activity. The functionality-driven optimization proposed here is not inherently linked to UAE; instead, it may be applied to any extraction technique, including UAE.

To test this approach, a green ultrasound-assisted extraction (UAE) process was developed for the edible brown algae *Himanthalia elongata*, *Eisenia bicyclis*, *Sargassum fusiforme*, and *Laminaria ochroleuca*, selected for their commercial relevance and widespread consumption. This study therefore establishes a functionality-driven extraction optimization strategy as a green and efficient approach for the valorization of these four edible brown algae.

## 2. Results

### 2.1. Optimization of the UAE Variables

The experimental design for process optimization combined univariate and multivariate approaches. Variables related to the mass-to-solvent interaction (algae amount, solvent type, and solvent concentration) were evaluated through univariate analysis. Ultrasound-dependent parameters (time and power) were optimized using a multivariate approach. Finally, a univariate study was performed to assess the effect of extraction temperature on the antioxidant activity of the extracts.

Initial extraction conditions were set at 20 mL of 50% ethanol, 5 min of sonication at 150 W, and 25 °C. These settings were initially used for a preliminary screening step, as commonly applied in the literature for brown macroalgae [12,15], to allow cross-species comparison before individual optimization. These parameters were subsequently adjusted according to the variable under study, based on the statistical evaluation of the antioxidant activity obtained, selecting the most effective and environmentally favourable conditions for the subsequent experiments. All assays were conducted in triplicate for each algal species.

#### 2.1.1. Amount of Algae

**Figure 1:**
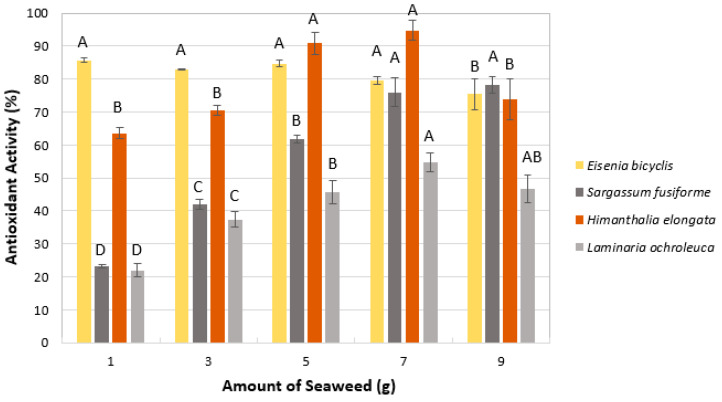
Effect of algae amount on antioxidant activity (RSA-DPPH). Mean ± SD, *n* = 3. Different letters (A–D) indicate significant differences (*p* < 0.05), Tukey’s HSD.

The effect of algae amount on antioxidant activity was evaluated between 1 and 9 g. As shown in Figure 1, all species except *E. bicyclis* displayed a similar trend: antioxidant activity increased as algae amount rose to 5–7 g, reaching approximately 54–95%, and then decreased at 9 g (46–78%). In contrast, *E. bicyclis* maintained high activity (80–85%) across 1–7 g, with no significant differences within this range (*p* > 0.05). The reduction in antioxidant activity at the highest biomass loading (9 g), observed in most species, may result from mass-transfer limitations and reduced cavitation efficiency when the suspension becomes too dense, hindering solvent penetration and slowing the diffusion of bioactive compounds. Based on these results and green extraction criteria, 1 g was selected as the optimal amount for *E. bicyclis*, 5 g for *H. elongata*, and 7 g for both *S. fusiforme* and *L. ochroleuca*.

#### 2.1.2. Type of Solvent

**Figure 2:**
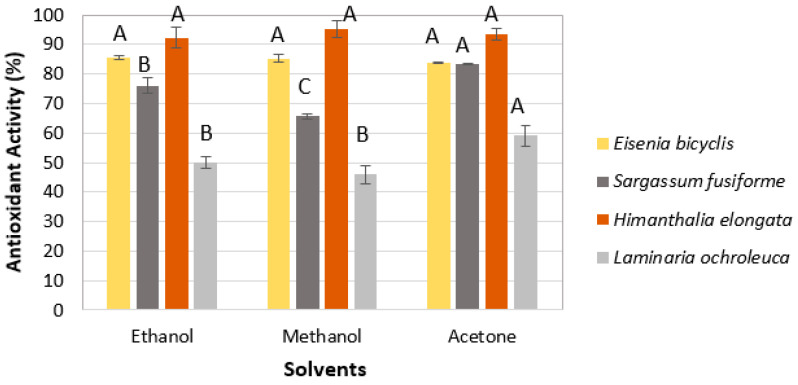
Effect of solvent type on antioxidant activity (RSA-DPPH). Mean ± SD, *n* = 3. Different letters (A–C) indicate significant differences (*p* < 0.05), Tukey’s HSD.

The effect of solvent polarity on antioxidant activity was evaluated using 50% (*v*/*v*) ethanol, methanol, and acetone (Figure 2). *E. bicyclis* and *H. elongata* showed no significant differences among solvents (*p* > 0.05), and ethanol was selected for both species due to its lower toxicity and suitability for green extraction. In contrast, *S. fusiforme* and *L. ochroleuca* exhibited significantly higher antioxidant activity when acetone was used, which was therefore selected for these two species. Although acetone was selected due to its superior extraction performance for these species, its use in downstream applications (e.g., food or cosmetics) would require complete removal or substitution with greener solvents.

#### 2.1.3. Solvent Concentration

**Figure 3:**
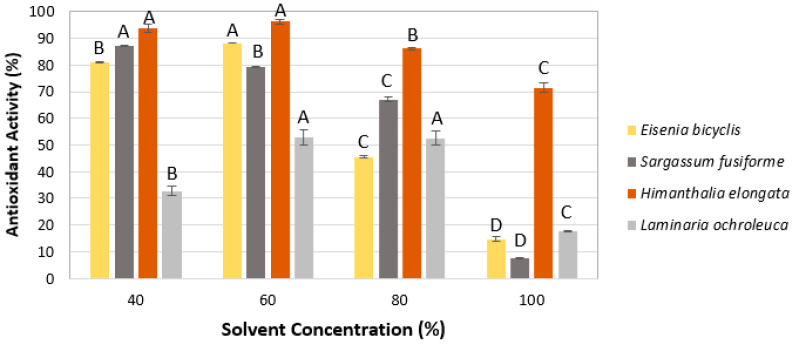
Effect of solvent concentration on antioxidant activity (RSA-DPPH). Mean ± SD, *n* = 3. Different letters (A–D) indicate significant differences (*p* < 0.05), Tukey’s HSD.

The effect of solvent concentration on antioxidant activity was evaluated using the solvents selected in the previous step (ethanol for *E. bicyclis* and *H. elongata*, and acetone for *S. fusiforme* and *L. ochroleuca*), ranging from 40% to 100% (*v*/*v*). As shown in Figure 3, intermediate solvent concentrations (40–60%) resulted in the highest antioxidant activities for all species (53–96% RSA-DPPH), whereas higher concentrations substantially reduced activity, particularly at 100% solvent, where values dropped to 7–71% RSA-DPPH. Accordingly, and as part of our green extraction criteria, the less resource-demanding condition within the highest statistically equivalent group was selected. Therefore, 40% was selected for *H. elongata* and *S. fusiforme*, and 60% for *E. bicyclis* and *L. ochroleuca*.

#### 2.1.4. Ultrasound Extraction Time and Power

**Figure 4:**
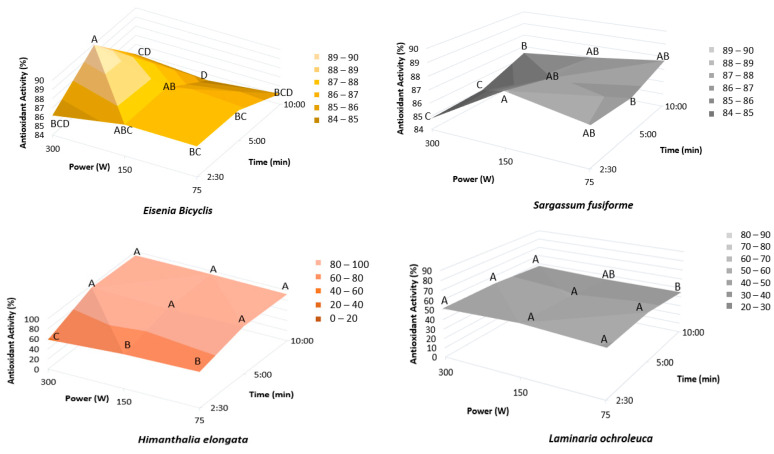
Effect of ultrasound time and power on antioxidant activity (RSA-DPPH). Mean of triplicates. Different letters (A–D) indicate significant differences (*p* < 0.05), Tukey’s HSD.

To evaluate the combined effect of extraction time and ultrasound power on antioxidant activity, different combinations of both variables were tested simultaneously. The conditions assessed covered a range from 2.5 to 10 min for extraction time and 75 to 300 W for ultrasound power. The results obtained (Figure 4) revealed distinct response patterns depending on the algal species. In *E. bicyclis* and *S. fusiforme*, antioxidant activity was mainly influenced by ultrasonic power, with higher values at intermediate to high levels. In contrast, *H. elongata* was more affected by extraction time, showing increased activity at intermediate to longer treatments. For *L. ochroleuca*, neither variable introduced significant differences among the tested conditions (*p* > 0.05).

Following our green extraction criteria, the lowest time and power conditions among those yielding statistically equivalent antioxidant activity were selected. Accordingly, the optimal settings were: 150 W for 2 min 30 s in *E. bicyclis*; 75 W for 2 min 30 s in *S. fusiforme*; 75 W for 5 min in *H. elongata*; and 75 W for 2 min 30 s in *L. ochroleuca*.

#### 2.1.5. Temperature

**Figure 5:**
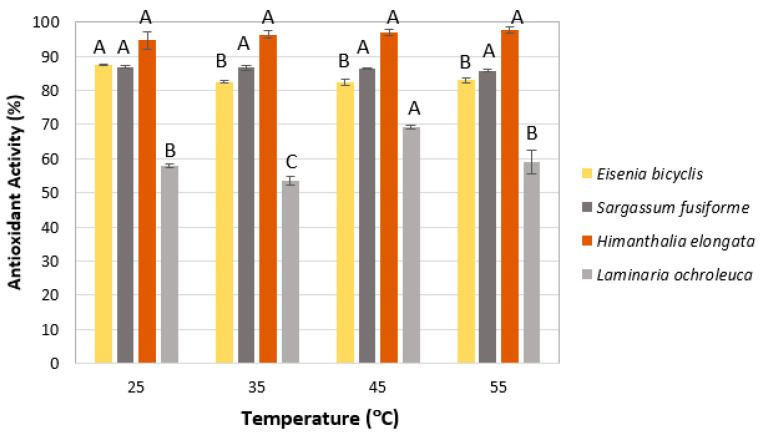
Effect of temperature on antioxidant activity (RSA-DPPH). Mean of triplicates. Different letters (A–C) indicate significant differences (*p* < 0.05), Tukey’s HSD.

The effect of temperature on the resulting antioxidant activity was evaluated at 25, 35, 45, and 55 °C. As shown in Figure 5, temperature did not significantly influence the antioxidant activity of *E. bicyclis*, *S. fusiforme*, or *H. elongata* (*p* > 0.05), which remained high within the range of approximately 87–98%. In contrast, *L. ochroleuca* exhibited a pronounced increase at 45 °C, reaching nearly 70% RSA-DPPH, while remaining below 60% at the other temperatures tested. Based on these results, the extraction temperature was set at 25 °C for all species except *L. ochroleuca*, for which 45 °C was selected as the optimal condition.

#### 2.1.6. Validation of Antioxidant Activity

After optimization of the extraction variables, the resulting antioxidant activities ranged from 69.17 ± 1.62 to 94.68 ± 0.06% RSA-DPPH (Table 1), with *Himanthalia elongata* showing the highest values, followed by *Eisenia bicyclis* and *Sargassum fusiforme*, while *Laminaria ochroleuca* exhibited the lowest activity.

To further validate these results, two additional antioxidant assays—ORAC and FRAP—were performed on the optimized extracts. These assays confirmed that *E. bicyclis* and *H. elongata* were the species with the highest antioxidant capacity. However, unlike RSA-DPPH, *E. bicyclis* showed the highest activity in both ORAC and FRAP.

### 2.2. Chemical Characterization of the Optimized Extracts

#### 2.2.1. Determination of Polyphenols by UHPLC-MS/MS

**Figure 6:**
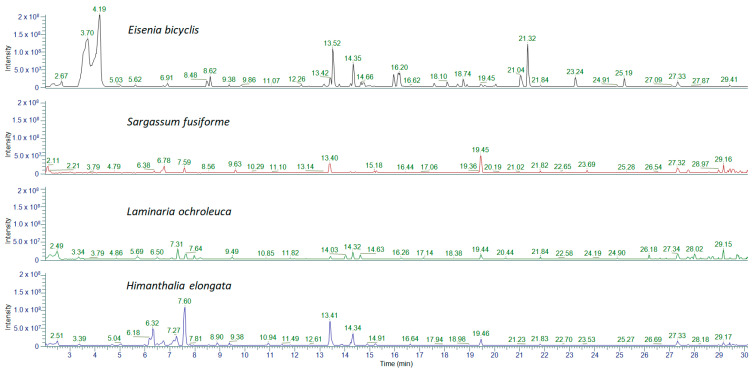
UHPLC-MS/MS chromatograms of the optimized extracts.

From the chromatographic analysis (Figure 6 and Table 2), clear differences in phenolic composition were observed. In *Eisenia bicyclis*, both phenolic acids and phlorotannins such as dieckol, eckol and 7-phloroeckol were detected, with the latter occurring at remarkably high concentrations compared to the other compounds identified. In the remaining algal species, these phlorotannins were absent, and only phenolic acids—mainly 2,3-dihydroxybenzoic acid, 2,4,6-trihydroxybenzoic acid, 2,5-dihydroxybenzoic acid, and 3,4-dihydroxybenzoic acid—were found, at concentrations that varied depending on the species.

#### 2.2.2. Determination of Pigments by HPLC-Fl

The results of the chromatographic analysis of the optimized extracts are shown in Table 3. All extracts contained Chlorophyll *a*, which was more abundant in *Eisenia bicyclis* than in the other algae studied. Lutein was also detected at higher levels in the *Eisenia bicyclis* extract, whereas it was not detectable in *Sargassum fusiforme*. Finally, fucoxanthin and violaxanthin were present at higher concentrations in the *Laminaria ochroleuca* extract compared with the other species analyzed.

## 3. Discussion

### 3.1. Optimization of the Ultrasound-Assisted Extraction Process

In this study, ultrasound-assisted extraction (UAE) was developed as a green alternative to conventional methods such as Soxhlet or cold percolation [10,21], due to its lower solvent and energy consumption, shorter processing time, and higher recovery efficiency. Similar advantages have been consistently reported by other authors [22,23,24,25].

Unlike most UAE optimizations that aim to maximize extraction yield or total phenolic content (TPC) [26,27,28], this study focused on maximizing functional antioxidant activity. This approach is especially relevant for brown macroalgae, where the relationship between TPC and antioxidant capacity remains controversial. Several studies have shown that antioxidant potential depends mainly on molecular structure: the number and position of hydroxyl groups and the degree of polymerization determine radical-scavenging ability. Shibata et al. (2008) and Yotsu-Yamashita et al. (2013) reported large activity differences among isolated phlorotannins from *Ecklonia* species [17,18], and Hermund et al. (2018) found that lower-molecular-weight phlorotannins from *Fucus vesiculosus* were more active than polymerized forms [16]. Likewise, Airanthi et al. (2011) and Maadane et al. (2015) observed weak correlations between TPC and antioxidant response, indicating contributions from pigments and polysaccharides [19,29].

Using this functionality-based optimization, extracts reached 69–98% RSA-DPPH, values comparable to those reported for *Fucus vesiculosus* (~90%) [26] and higher than those obtained with conventional extraction of *Himanthalia elongata* (~76%) [30]. The effect of biomass agrees with reports highlighting the importance of the sample-to-solvent ratio [27,28]. Solvent polarity was also critical: ethanol gave the best results for *Eisenia bicyclis* and *Himanthalia elongata*, and acetone for *Sargassum fusiforme* and *Laminaria ochroleuca*, with optimal concentrations of 40–60%. This agrees with previous findings showing that solvents of intermediate polarity, especially aqueous ethanol or acetone, enhance phenolic extraction [26,27,31,32]. In our case, this effect was clearly reflected in the composition and functionality of the optimized extracts: intermediate ethanol/water or acetone/water mixtures enabled the simultaneous extraction of phenolic compounds and pigments, resulting in the highest antioxidant activities. However, the optimal polarity differed between species. In *E. bicyclis*, 60% ethanol was required to maximize activity, likely due to the presence of abundant, less polar phlorotannins, which are efficiently solubilized at lower polarity together with carotenoids. In contrast, *H. elongata* reached similarly high activities with 40% ethanol, consistent with a phenolic profile characterized by more polar compounds.

Ultrasonic parameters and temperature showed species-dependent effects. Extraction time most affected *Himanthalia elongata*, while power had a moderate influence in *Eisenia bicyclis* and *Sargassum fusiforme* and was negligible in *Laminaria ochroleuca*. These findings are consistent with reports linking UAE efficiency to algal cell-wall composition and solvent accessibility [28,33]. In our study, *E. bicyclis* was the only species for which a higher ultrasonic power and a slightly more ethanolic solvent concentration were required to reach optimal extraction performance. This distinctive behavior aligns with its exceptionally dense and highly cross-linked cell-wall matrix—rich in alginate and cellulose—which makes *E. bicyclis* the most structurally rigid among the species evaluated, thereby hindering cavitation-driven disruption and slowing mass transfer during UAE [34].

Finally, for *Laminaria ochroleuca*, activity increased at 45 °C, in agreement with previous studies indicating that moderate temperatures (40–60 °C) improve diffusion without compound degradation [35,36]. On the contrary, higher-energy methods such as MAE or PLE can further enhance phenolic recovery at elevated temperatures [37,38], confirming that optimal temperature is both technique- and species-dependent.

### 3.2. Comparative Antioxidant Activity

After optimization through the DPPH assay, all extracts displayed high radical-scavenging activity (69–95%), with *Himanthalia elongata* showing the highest response, followed by *Eisenia bicyclis*, *Sargassum fusiforme*, and *Laminaria ochroleuca*. Validation with ORAC and FRAP assays confirmed the overall trend, although the ranking slightly changed—*E. bicyclis* was most active by ORAC and FRAP, followed by *H. elongata*, while *S. fusiforme* and *L. ochroleuca* remained less active.

Such differences are expected, as each assay targets distinct antioxidant mechanisms: DPPH mainly reflects hydrogen- or single-electron transfer to a stable organic radical, ORAC quantifies peroxyl-radical scavenging under kinetic control, and FRAP measures reducing capacity toward ferric ions. Consequently, responses may vary depending on the predominant antioxidant mechanism in each extract.

Discrepancies among assays have been frequently reported for brown macroalgae. Agregán et al. (2018) observed high activities across DPPH, ORAC, and FRAP for *Bifurcaria bifurcata*, *Ascophyllum nodosum*, and *Fucus vesiculosus*, though rankings differed between methods [39]. Silva et al. (2021) also found stronger correlations between TPC and FRAP than with DPPH, emphasizing the influence of solvent polarity [40].

In our case, the differences observed between *E. bicyclis* and *H. elongata* can be explained by the polarity of the optimized solvent mixtures and the resulting phenolic profiles. The 40% ethanol–water system favored the extraction of more polar, low-molecular-weight hydroxybenzoic acids in *H. elongata*, which may react rapidly with DPPH, consistent with its slightly higher %RSA-DPPH. In contrast, the 60% ethanol–water mixture used for *E. bicyclis* enriched the extract in highly hydroxylated, higher-molecular-weight phlorotannins such as dieckol and eckol. These compounds display multiple ortho-dihydroxyl groups and a greater degree of oligomerization, structural features that enhance both electron-transfer and hydrogen-atom-transfer reactions, explaining the superior activity of *E. bicyclis* under FRAP and ORAC conditions despite its similar DPPH response.

Therefore, a positive qualitative correlation was observed among the three antioxidant assays, particularly for *S. fusiforme* and *L. ochroleuca*, which maintained consistent activity rankings. In contrast, the slight differences detected for *E. bicyclis* and *H. elongata* between DPPH and ORAC/FRAP reflect the specific antioxidant mechanisms probed by each method, as well as the polarity of the optimized solvents and the structural features of the compounds extracted.

In microalgae, Goiris et al. (2012) showed that phenolics and carotenoids jointly determine antioxidant capacity, explaining the inconsistent agreement between DPPH and FRAP/TEAC assays [41]. Yuan et al. (2018) likewise reported stronger correlations between FRAP and TPC (r ≈ 0.78), whereas DPPH and ABTS gave divergent rankings, confirming the assay-dependent nature of antioxidant assessment [37].

Despite these differences, all methods consistently identified *Eisenia bicyclis* and *Himanthalia elongata* as the most antioxidant species, supporting their potential as promising sources for industrial or biotechnological applications.

### 3.3. Characterization of Antioxidant Compounds

Chromatographic analysis of the optimized extracts provided further insight into the compounds responsible for the observed activity. *Eisenia bicyclis* showed the highest antioxidant capacity, consistent with its high content of tannin-type phlorotannins such as dieckol, along with phenolic acids also detected in the other species. Conversely, the low activity of *Laminaria ochroleuca* may be explained by its lower phenolic levels.

For *Himanthalia elongata* and *Sargassum fusiforme*, the phenolic composition also supported the antioxidant trends observed. *H. elongata* exhibited a higher concentration of 2,4,6-trihydroxybenzoic acid, a more hydroxylated phenolic structure than that found in *S. fusiforme,* which is consistent with its superior radical-scavenging activity. This agrees with previous studies showing that the antioxidant performance of phenolic compounds depends not only on abundance but also on structural features such as the number and position of hydroxyl (–OH) groups, which determine both radical-scavenging and metal-chelating capacity [19,23,42]. Accordingly, the overall antioxidant behavior observed in this study can be explained by the relative presence of highly hydroxylated phenolic structures, being more pronounced in *E. bicyclis*, followed by *H. elongata*, and comparatively lower in *S. fusiforme* and *L. ochroleuca*.

The pigments present in the optimized extracts also contributed to their antioxidant behavior. *Eisenia bicyclis* showed the highest levels of Chlorophyll *a* and lutein, whereas *Laminaria ochroleuca* exhibited greater amounts of carotenoids such as fucoxanthin and violaxanthin. These pigment profiles reflect the selectivity of the extraction solvents rather than the native composition of the algae, since solvent polarity strongly influences pigment recovery—aqueous ethanol favoring chlorophylls and xanthophylls, and acetone mixtures enhancing carotenoid extraction [43,44].

Although both chlorophylls and carotenoids exhibit antioxidant activity, their mechanisms differ: carotenoids (notably fucoxanthin) mainly quench singlet oxygen and peroxyl radicals, whereas chlorophylls and xanthophylls act through radical-scavenging and metal-chelating mechanisms [32,41]. However, in our study the extract from *L. ochroleuca*, which was particularly rich in carotenoids, remained less active, indicating that carotenoids alone cannot offset a low phenolic abundance.

This observation is consistent with previous reports showing that high carotenoid contents in brown algae do not always translate into superior antioxidant activity, and that phenolic compounds often play a predominant role [45,46].

In agreement with this interpretation, the high antioxidant capacity of *E. bicyclis* is plausibly linked to its remarkable content of high-molecular-weight phlorotannins—particularly dieckol, eckol, and 7-phloroeckol—together with relevant levels of Chlorophyll *a* and lutein. In *L. ochroleuca*, the comparatively lower activity is consistent with its reduced phenolic content, despite a higher proportion of carotenoids such as fucoxanthin and violaxanthin. Similarly, in *H. elongata* and *S. fusiforme*, the antioxidant response appears to be more attributable to their phenolic composition than to their pigment content.

Therefore, follow-up fractionation experiments separating pigment-rich and polyphenol-rich fractions, and testing them independently and in combination, will be required to directly evaluate potential synergistic interactions in our optimized extracts.

Overall, these findings confirm the efficiency of UAE as a green extraction method for brown algae and highlight the importance of optimizing process variables based on extract functionality rather than yield.

## 4. Materials and Methods

### 4.1. Sample Collection

*Himanthalia elongata* and *Laminaria ochroleuca* were purchased from Algamar (Pontevedra, Spain), while *Sargassum fusiforme* and *Eisenia bicyclis* were obtained from Terra Verda^®^ (Herbolarios Navarro, Valencia, Spain). The first two species were harvested on the Galician north coast (northwestern Spain), and the latter two originated from Japan. All algae were dehydrated at low temperature and partially cut prior to distribution. A single batch of each species was used. Samples were ground, sieved, and the <500 μm fraction was selected to ensure sample homogeneity. The powdered algae were stored in airtight bags and kept in a desiccator until the extraction process.

### 4.2. Ultrasound-Assisted Extraction (UAE)

Ultrasound-assisted extraction was performed in an ultrasonic bath (ATM10L, Ovan, Barcelona, Spain) operating at a fixed frequency of 40 kHz and a nominal power of 300 W (10 L capacity), corresponding to an estimated acoustic power density of ~30 W L^−1^. The system is equipped with a microprocessor-controlled thermostat and digital temperature display, enabling continuous monitoring and regulation of the extraction temperature. For the optimization experiments, the following extraction variables were evaluated: algae amount (1–9 g), solvent type (ethanol, methanol, and acetone; all at 50% *v*/*v*), solvent concentration (40–100% *v*/*v*), ultrasound power (75–150 W), extraction time (2.5–10 min), and temperature (25–45 °C). The solvent volume was kept constant (20 mL) in all assays.

Solvent type, solvent concentration, algae amount, and temperature were optimized using a univariate approach, whereas ultrasound power and extraction time were assessed jointly to account for their interactive effects on cavitation efficiency. After optimization, UAE was performed using the following conditions established for each species: *Eisenia bicyclis* (1 g, ethanol/water 60:40 *v*/*v*, 150 W, 2.5 min, 25 °C), *Sargassum fusiforme* (7 g, acetone/water 40:60 *v*/*v*, 75 W, 2.5 min, 25 °C), *Himanthalia elongata* (5 g, ethanol/water 40:60 *v*/*v*, 75 W, 5 min, 25 °C), and *Laminaria ochroleuca* (7 g, acetone/water 60:40 *v*/*v*, 75 W, 2.5 min, 45 °C).

After extraction, the mixtures were centrifuged at 4000 rpm for 10 min, and the supernatants were filtered through 0.45 μm nylon membranes prior to analysis. All extractions were performed in triplicate, and the resulting extracts were analysed on the same day.

### 4.3. Antioxidant Activity

#### 4.3.1. DPPH Free Radical Scavenging Activity (DPPH-RSA)

The study of the antioxidant activity was carried out using the DPPH method with some modifications. The molecule 2,2-diphenyl, 1-picrylhydrazyl (DPPH) is a free, stable, reducing system, which produces a colour change from purple to yellow, observable in an absorption spectrum at a wavelength of 515 nm [47]. In brief, 500 μL of extract was placed in a test tube to which 5 mL of 0.1 mM DPPH was added and kept for 20 min in the dark at room temperature. Antioxidant activity was determined by recording the absorbance at a wavelength of 515 nm. The blank and the DPPH control solution were prepared in methanol. The results of radical scavenging activity (RSA) are expressed in % and determined from the following formula:

$$DPPH-RSA(\%)=\frac{Acontrol-Asample}{Acontrol}\times 100$$

#### 4.3.2. Oxygen Radical Absorbance Capacity (ORAC) Assay 

The ORAC assay measures the oxidative degradation of the fluorescent probe fluorescein caused by peroxyl radicals, resulting in a time-dependent loss of fluorescence [48]. All solutions were prepared in phosphate buffer. The standard curve was obtained from Trolox solutions (5 μM–1 mM), and the optimized extracts were diluted 1:100.

Reactions were performed in triplicate in black 96-well microplates by adding 25 μL of each Trolox standard (including blank) and the diluted extracts to separate wells, followed by 150 μL of 1 μM fluorescein. Plates were incubated at 37 °C in the dark for 30 min. The oxidative reaction was then initiated by adding 25 μL of 2,2′-azobis(2-methylpropionamidine) dihydrochloride (AAPH, 50 mg mL^−1^). Fluorescence decay was recorded every 30 s for 90 min at 485 nm excitation and 518 nm emission using a multimode microplate reader (Varioskan Lux, Thermo Fisher, Waltham, MA USA).

Results were calculated from the regression equation lg(y) = 0.5556 lg(x) + 4.7373 (R^2^ = 0.997) and expressed as μmol Trolox equivalents per gram of dry weight (μmol TE g^−1^ DW).

#### 4.3.3. Ferric Reducing Antioxidant Power (FRAP) Assay 

The FRAP assay is a colorimetric method that quantifies the reduction of ferric (Fe^3+^) to ferrous (Fe^2+^) ions by antioxidants under acidic conditions, resulting in the formation of an intense blue Fe^2+^–TPTZ complex [49]. The standard curve was prepared using ferrous ammonium sulfate solutions (0.2–2 mM) in buffer at pH 3.6. A positive control was included to verify assay performance, and background correction was applied to account for sample interferences.

The reaction mixture, consisting of the test buffer, FeCl_3_ solution, and ferric probe, was freshly prepared. In a flat-bottom 96-well microplate, 10 μL of each standard (including blank) and the diluted algal extracts (1:5, *v*/*v*) were added to separate wells, followed by 190 μL of the reaction mixture. Absorbance was recorded at 594 nm every minute for 60 min at 37 °C using a multimode microplate reader (Varioskan Lux, Thermo Fisher). Results were calculated from the standard curve (y = 1895.68x − 17.7216, R^2^ = 0.999) and expressed as μmol Fe^2+^ equivalents per gram of dry weight (μmol Fe^2+^ g^−1^ DW).

### 4.4. Chromatographic Analysis

#### 4.4.1. Polyphenol Analysis (UHPLC/MS-MS)

Phenolic compound quantification was performed using the IBMCP Metabolomics Platform (UPV-CSIC, Valencia, Spain).

1 µL of each extract was injected into a Orbitrap Exploris 120 mass spectrometer coupled with a Vanquish UHPLC System (Thermo Fisher Scientific, Waltham, MA, USA). Analysis was carried out by reverse-phase ultraperformance liquid chromatography using a Acquity PREMIER BEH C18 UPLC column (1.7 uM particle size, dimensions 2.1 × 150 mm) (Waters Corp., Mildford, MA, USA).

The mobile phase consisted of 0.1% formic acid in water (phase A), and 0.1% formic acid in acetonitrile (phase B). The solvent gradient process was performed as follows: 0.5% solvent B over the first 2 min, 0.5–30% solvent B over 25 min, 30–100% solvent B over 13 min, 2 min at 100% B, return to the initial 0.5% solvent B over 1 min, and conditioning at 0.5% B for 2 min. The flow rate was 0.4 mL/min and the injection volume was 1 µL. The column temperature was set at 40 °C.

Ionisation was performed with heated electrospray ionization (H-ESI) in positive and negative mode. Samples were acquired in full scan mode (resolution set at 120,000 measured at FWHM). For absolute quantification, calibration curves were established using authentic standards, with genistein employed as the internal standard. For semi-quantitative analysis, calibration curves of the most structurally related authentic compounds were applied, and the results were expressed as equivalents of the corresponding standard. Data processing was performed with TraceFinder software version 5.1 (Thermo Scientific, Waltham, MA, USA). Three replicates were analysed per sample.

#### 4.4.2. Pigment Analysis (HPLC/Fl)

Chromatographic analyses were performed using an HPLC system (Hitachi 890-0442, Tokyo, Japan) equipped with a fluorescence detector (L-2480), column oven (L-2300), autosampler (L-2200U), and pump (L-2130), controlled by EZChrom Elite™ software, version 3.3.2. Separation was achieved on a LiChrospher^®^ 100 RP-18 reversed-phase column (250 × 4 mm, 5 µm; Darmstadt, Germany).

For pigment determination, 60 µL of each extract were injected at 30 °C. The mobile phase consisted of methanol (A) and water (B) at a flow rate of 1.0 mL min^−1^, using the following gradient: 90% A/10% B for 5 min, followed by 100% A/0% B for 25 min. Pigments were identified according to their retention times (t_r_) and quantified at their optimal excitation and emission wavelengths (λ_ex_/λ_em_) recorded by the fluorescence detector. Calibration curves were prepared for each compound within the following linearity ranges: fucoxanthin (6–40 µg mL^−1^), violaxanthin (0.75–10 µg mL^−1^), lutein (0.75–10 µg mL^−1^), and Chlorophyll *a*’ (0.25–10 µg mL^−1^). All analyses were performed in triplicate.

### 4.5. Statistical Analysis

All analyses were performed in triplicate for each parameter and variable studied. Data are expressed as mean ± SD. Normality (Shapiro–Wilk test) and homoscedasticity were evaluated (*p* > 0.05), applying arcsine square root transformation when required. Differences among groups were analyzed by one-way ANOVA followed by Tukey’s HSD test (*p* < 0.05). Statistical analyses were conducted using IBM SPSS Statistics 23.0 (Windows, version 2021).

## 5. Conclusions

This study developed and optimized a green ultrasound-assisted extraction (UAE) methodology for four edible brown algae, prioritizing the functional antioxidant response of the extracts rather than phenolic yield or total phenolic content. The process, based on mild and energy-efficient conditions and reduced solvent consumption, yielded highly active extracts (69.17–94.68% RSA-DPPH) while aligning with the principles of green chemistry.

Complementary antioxidant assays (ORAC and FRAP) confirmed the robustness of the optimization strategy, consistently identifying *Eisenia bicyclis* and *Himanthalia elongata* as the most antioxidant species.

Chromatographic analysis revealed that the strong antioxidant behavior observed in the optimized *Eisenia bicyclis* extract is mainly attributed to its high content of tannin-type phlorotannins, together with chlorophyll- and xanthophyll-type pigments (such as lutein), which provide complementary radical-scavenging mechanisms.

Overall, this work highlights the relevance of a functionality-driven optimization strategy to maximize the bioactivity of marine algal extracts using a rapid, sustainable methodology. The optimized extracts, particularly from *E. bicyclis* and *H. elongata*, show potential for future valorization at the pilot scale. Further studies, including fractionation experiments, will be required to confirm the contribution and possible synergy of the main antioxidant components and to support future scale-up efforts.

## Figures and Tables

**Table 1:** Antioxidant activity of the optimized extracts determined by DPPH, ORAC and FRAP tests.

Algae	DPPH (% ± σ)	ORAC (μmol TE g^−1^ DW ± σ) ^1^	FRAP (µmol Fe^+2^ g^−1^ DW ± σ) ^2^
*Eisenia bicyclis*	87.43 ± 0.26	491.30 ± 17.42	87.65 ± 4.29
*Sargassum fusiforme*	86.97 ± 0.27	20.75 ± 0.26	7.64 ± 0.47
*Himanthalia elongata*	94.68 ± 0.06	114.39 ± 4.15	36.2 ± 2.40
*Laminaria ochroleuca*	69.17 ± 1.62	18.63 ± 1.51	1.24 ± 0.11

^1^ µmol Trolox Equivalents g^−1^ dry weight. ^2^ µmol Fe^+2^ Equivalents g^−1^ dry weight.

**Table 2:** Chromatographic analysis of polyphenols in the optimized extracts (*n* = 3).

Compound	*t_R_* (min)	*E. bicyclis* (ng g^−1^ DW)	*S. fusiforme* (ng g^−1^ DW)	*H. elongata* (ng g^−1^ DW)	*L. ochroleuca* (ng g^−1^ DW)
2,3,4-trihydroxybenzoic acid ^b^	5.57	<LOD	22.11 ± 1.68	<LOD	<LOD
2,3-dihydroxybenzoic acid ^b^	10.15	64.14 ± 4.15	87.62 ± 7.48	278.91 ± 16.22	46.29 ± 1.54
2,4,6-trihydroxybenzoic acid ^a^	4.78	2672.54 ± 116.00	189.93 ± 16.07	475.09 ± 28.95	125.87 ± 9.85
2,5-dihydroxybenzoic acid ^b^	8.02	<LOD	31.06 ± 2.28	9.45 ± 0.24	5.63 ± 0.35
3,4-dihydroxybenzoic acid ^a^	5.65	404.55 ± 9.94	813.52 ± 61.60	438.34 ± 29.78	625.75 ± 25.77
3-hydroxybenzoic acid ^b^	10.48	<LOD	40.57 ± 1.52	<LOD	34.27 ± 2.55
2-phloroeckol *^c^ (×10^3^)	14.96	1.37 ± 0.08	<LOD	<LOD	<LOD
7-phloroeckol *^c^ (×10^3^)	14.76	124.28 ± 7.33	<LOD	<LOD	<LOD
Dieckol ^a^ (×10^3^)	21.32	129.85 ± 64.76	<LOD	<LOD	<LOD
Eckol *^c^ (×10^3^)	14.35	534.61 ± 18.71	<LOD	<LOD	<LOD

* Tentative identification. ^a^ absolute quantification. ^b^ semi-quantitative (2,4,6-trihydroxybenzoic acid equivalents). ^c^ semi-quantitative (dieckol equivalents).

**Table 3:** Chromatographic analysis of pigments by HPLC-Fl in the optimized extracts (*n* = 3).

Compound	t_R_^a^ (min)	λ_exc._^b^ (nm)	λ_em._^c^ (nm)	*E. bicyclis* (ng g^−1^ DW± σ)	*S. fusiforme* (ng g^−1^ DW± σ)	*H. elongata* (ng g^−1^ DW± σ)	*L. ochroleuca* (ng g^−1^ DW± σ)
Fucoxanthin	10.1	461	515	5.35 ± 0.20	<LOD	0.96 ± 0.02	18.70 ± 0.01
Violaxanthin	11.03	472	512	<LOD	<LOD	<LOD	0.20 ± 0.01
Lutein	13.23	472	512	4.17 ± 0.01	<LOD	0.50 ± 0.04	0.28 ± 0.01
Chlorophyll *a*	27.01	430	675	4.13 ± 0.25	0.27 ± 0.01	0.70 ± 0.07	1.23 ± 0.17

^a^ Retention time. ^b^ Excitation wavelength. ^c^ Emission wavelength.

## Data Availability

Data are available on reasonable request from the corresponding author (carolina.padron@ucv.es).

## Abbreviations

The following abbreviations are used in this manuscript:

UAE	Ultrasound-Assisted Extraction
DPPH	2,2-diphenyl, 1-picrylhydrazyl
ORAC	Oxygen Radical Absorbance Capacity
FRAP	Ferric Reducing Antioxidant Power
TPTZ	2,4,6-tripyridyl-s-triazine
